# Monocyte Adhesion Molecules Expression in Patients with Chronic Hepatitis C Liver Disease

**DOI:** 10.4084/MJHID.2013.054

**Published:** 2013-09-02

**Authors:** Nora E.I. El-Bassiouni, Ola M. Mahmoud, Eman G El Ahwani, Raafat A. Ibrahim, Azza E.I. El Bassiouny

**Affiliations:** 1 Department of Haematology, Theodor Bilharz Research Institute, Imbaba, Giza, Egypt.; 2 Department of Immunology, Theodor Bilharz Research Institute, Imbaba, Giza, Egypt.; 3 Department of Hepato-Gastroenterology, Theodor Bilharz Research Institute, Imbaba, Giza, Egypt.

## Abstract

**Background:**

Chronic viral hepatitis is histologically characterized by predominantly periportal infiltration of mononuclear cells, including lymphocytes and monocytes/macrophages. Intralobular infiltration of these inflammatory cells is an ominous sign of deterioration and a criterion for disease activity.

**Objective:**

To assess the monocyte inflammatory milieu, monocytes adhesion molecules, their endothelial receptors, cytokines and chemokines in patients with HCV induced chronic liver disease, in an attempt to clarify the role of blood monocytes in induction of inflammation and fibrogenesis in chronic hepatitis C liver disease.

**Subjects and Methods:**

The current study included 60 patients with chronic liver disease categorized into 2groups: Patients chronic hepatitis C (CHC) and patients with liver cirrhosis (LC), 15 patients each; 15 healthy subjects were included as normal controls. Immunophenotype characterization was carried out by flowcytometric analysis for identification of CD11a, CD11b and CD49d monocyte surface antigen expression in different groups studied. The circulating levels of the soluble adhesion molecules (sE-selectin, sICAM-1 and sVCAM-1), cytokines (TNF-α and IL-1) and chemokines (MCP-1) were also assessed by immunoassays.

**Results:**

Data demonstrated a significant increase (*p*<0.01) in the surface expression of CD11a on peripheral blood monocytes and in the circulating levels sE-selectins, sICAM-1, sVCAM-1 and TNF-α in both groups of patients compared to healthy subjects. Data also revealed a significant increase (*p*<0.01) in the surface expression of each of CD11b and CD49d on peripheral blood monocytes and in the circulating levels sICAM-1, sVCAM-1 and TNF-α in patients with LC compared to those with CHC. Moreover, data demonstrated that the increase in surface antigen expression of each CD11a (*p*<0.01), CD11b (*p*<0.05) and CD49d (*p*<0.01) on circulating peripheral blood monocytes is positively correlated with the increase in the circulating levels of each of sICAM-1 and sVCAM-1 in the both groups of patients.

**Conclusions:**

These findings suggest that the modulation of monocyte-subset recruitment into the liver via adhesion molecules or cytokines/cytokine receptors may represent promising approaches for therapeutic interventions in human liver fibrosis. Measurement of serum soluble adhesion molecules may be useful for monitoring progression of liver inflammation and fibrosis during CHC.

## Introduction

Hepatitis C virus (HCV) is considered the most common causes of chronic hepatitis in Egypt. Liver fibrosis is the scarring process that represents the liver’s response to injury. Over time this process can result in cirrhosis of the liver, which once have developed the serious complications of liver disease may occur including portal hypertension, liver failure and liver cancer. Cirrhosis and liver cancer are now among the top ten causes of death worldwide, and in many developed countries liver disease is now one of the top five causes of death in middle-age.^1^

Liver damage in chronic hepatitis C (CHC) is commonly attributed to immune-mediated mechanisms.^2^–^3^ Hepatic fibrosis is characterized by abnormal excessive accumulation of extracellular matrix accompanied by exaggerated cytokine release. Recruitment and trans-differentiation of peripheral blood cells, in particular, monocytes into injured liver^4^ may play a role in this respect. Chronic viral hepatitis is histologically characterized by predominantly periportal infiltration of mononuclear cells, including lymphocytes and monocytes/macrophages. Intralobular infiltration of these inflammatory cells is an ominous sign of deterioration and a criterion for disease activity.^5^

Monocytes circulate as nonadherent cells in the blood and migrate as adherent cells through tissues depending on the ordered expression of specific cell surface adhesion molecule (AM) on a variety of cells.^6^ Monocyte adhesion to intact or damaged vascular endothelium requires several families of AM, in particular, the selectin family for the initial contact of monocytes with the endothelium^7^ and the integrin receptors for the subsequent firm adhesion and transmigration.^8^ On monocyte, these include leukocyte function antigen-1 (LFA-1 or CD11a) and Mac-1 (CD11b) and their endothelial ligand intercellular AM (ICAM-1 or CD45). In addition, a very late antigen-4 (VLA-4 or CD49d) is a monocyte ligand that promotes monocyte adherence to endothelial vascular cell adhesion molecule-1 (VCAM-1) and to connective tissue components.^6^ ICAM-1 and VCAM-1 are strongly expressed on sinusoidal lining cells in chronic hepatic inflammation due to HCV infection and play a key role in leukocyte recruitment and extravasation.^9^ Moreover, it was found that HCV-infected hepatocytes but not normal hepatocytes express ICAM-1.^10^

Cytokines and chemoattractants direct leukocyte trafficking and positioning within tissues, bring flexibility and specificity to recruitment,^11^ initiate and regulate the margination and extravasation of monocytes. Tumor necrosis factor-α (TNF-α) and interleukin-1 (IL-1) induce leukocyte infiltration by up-regulating the expression of leukocyte AM on vascular endothelial cells.^12^ Monocyte chemoattractant protein-1 (MCP-1) plays an important role in the recruitment of monocytes and macrophages from the bloodstream to inflamed tissue. It is also implicated in the pathogenesis of a variety of diseases that are characterized by mononuclear cell infiltration and maintenance of the inflammatory infiltrate.^13^

We aim to analyze the monocyte inflammatory milieu, monocytes adhesion molecules, their endothelial receptors, cytokines and chemokines in patients with HCV induced chronic liver disease, in an attempt to clarify the role of blood monocytes in induction of inflammation and fibrogenesis in chronic hepatitis C liver disease.

## Subjects and Methods

### Patients

Seventy-five individuals, of whom sixty patients with HCV induced chronic liver disease admitted to the Hepato-Gastroenterology Department, Theodor Bilharz Research Institute, Imbaba, Giza, Egypt and 15 age- and sex-matched healthy adults were enrolled in this study. Patients have M/F ratio of 38/22 and mean age of 45.6 ± 6.4 (range 22–60 years). The controls comprised 10 males and 5 females having a mean age of 42.8 ± 6.6, their liver function tests were within normal range and had no serologic evidence of hepatitis B and/or C viruses. The study was approved by the Institution’s Human Research Ethics Committee.

Diagnosis and classification of patients were based on full medical history, thorough clinical examination, liver and kidneys function tests, parasitological examination, abdominal ultrasonography, procto-sigmoidoscopy and liver biopsies. None of the subjects gave history of medication with interferon and ribavirin or drugs known to have influence on the coagulation process within 8 weeks of study time or had a renal impairment based on normal creatinine clearance. Cases with schistosomiasis infection, chronic viral diseases other than HCV, nonalcoholic steatohepatitis, biliary disorders, and malignancies were excluded from the study. All studied patients had CHC infection with circulating anti-HCV antibodies and were categorized into 2 groups: patients with CHC and patients with liver cirrhosis (LC), 30 cases each.

### Serological tests

Hepatitis B markers, including hepatitis B surface antigen and anti-HBs antibodies, total and IgM class antibodies against hepatitis B core antigen, hepatitis B antigen, and anti-HBe antibodies, were tested using commercially available enzyme immunoassay kits (Abbott Laboratories; North Chicago, Illinois, USA). Circulating anti-HCV antibodies were detected using Murex enzyme immunoassay kit (Murex anti-HCV, Version V; Murex Diagnostics; Dartford, England). The presence of HCV-RNA in patients’ sera was detected by real-time polymerase chain reaction using the Amplicor test (Roche Diagnostic Systems; Meylan, France).

### Immunological studies

Serum levels of circulating sE-selectin, sICAM-1, sVCAM-1, TNF-α, IL-1 and MCP-1 were measured using respective ELISA commercial kit (R&D Systems Inc, Minneapolis, USA). The coefficient of variation between duplicate samples was less than 10%. Normal values were indicated for each assay by the purchasing company and were not significant key different from the values obtained from the 15 healthy subjects. The latter values were considered, therefore, as reliable control values.

### Flowcytometric analysis

Detection of circulating peripheral blood monocyte surface adhesion molecule antigens expression was performed by analysis of patients venous blood samples using mouse anti-human fluorescein isothiocyanate (FITC) conjugated CD11a (LFA-1) monoclonal antibody and phycoerytrin (PE) conjugated CD11b (Mac-1) and CD49d (VLA-4) monoclonal antibodies (Becton Dickinson, Mountain view, CA, USA) by flow cytometry (Coulter, Coultronic, Margency, France). The IgG1, IgG2a and IgG2b were used as isotype controls. Five thousand gated monocytes were counted. Cells expressing receptor for CD11a, CD11b and CD49d emitted fluorescence signals which would be summated and multiplied in the PMTs and the computer would analyze the data as a signal frequency histogram.

### Statistical analysis

The Statistical Package for Social Sciences (SPSS) for Windows (version 11) computer program was used for statistical analysis. Means of different groups were compared using one-way ANOVA. Comparison between percent positive cases was calculated by *Chi-square* test. A *p* value <0.05 was considered statistically significant. Pearson correlation coefficient *r* was used to measure the relationship between 2 variables.

## Results

Progressive impairment of liver function tests, such as the increase in the activity of aminotransferases and bilirubin concentrations, were observed to increase together with the advancement of liver cirrhosis (Table 1). A decrease in albumin concentrations concomitant with an increase in prothrombin time was noted in the diseased groups compared to controls, and the alterations in these parameters paralleled the progress of the disease.

### Surface expression of monocyte adhesion molecule

Flow cytometric analysis revealed (Table 2) increased surface antigen expression of LFA-1 (CD11a) and Mac-1 (CD11b) and VLA-4 (CD49d) on circulating peripheral blood monocytes in patients with HCV which match the advancement of the disease. A significant increase in surface expression of CD11a on circulating peripheral blood monocytes was noticed in patients with CHC (*p*<0.05) and LC (*p*<0.01) compared to controls. Similarly, an increase in surface expression of CD11b (*p*<0.05) and CD49 (*p*<0.01) on circulating peripheral blood monocytes was also found in patients with LC compared to control group. Moreover, a marked increase (*p*<0.01) in surface expression of CD11b and CD49d on circulating peripheral blood monocytes was detected in patients with LC compared to those with CHC.

### Circulating levels of soluble adhesions molecules

Data also revealed (Table 2) a statistically significant increase (*p*<0.01) in the levels of soluble adhesion molecules sE-selectin, sICAM-1 and sVCAM- both diseased groups compared to controls. Moreover, a statistically significant increase (*p*<0.01) in the levels of sICAM-1 and sVCAM was detected in patients with LC compared to those with CHC revealing that the increase in the levels of these soluble adhesion molecules parallels the disease progression.

### Circulating levels of cytokines

A statistically significant increase (*p*<0.01) in circulating levels of TNF-α was found in both groups of patients compared to controls and in patients with LC compared to those with CHC (Table 2). Data revealed no statistically significant difference in the levels of IL-1 level in different groups studied. Although an increase in the levels of MCP-1 was found in patients with CHC and LC compared to controls and in LC group compared to patients with CHC, data were comparable.

### Correlation analysis

Correlations analyses between different studied parameters were demonstrated (Table 3).

## Discussion

The hepatic vascular bed is a unique low-flow environment through which leukocytes are recruited to the liver during homeostatic immune surveillance and in response to infection or injury. The rate of leukocyte recruitment and the nature of cells recruited through the sinusoids in response to inflammatory signals will shape the severity of disease.^11^ Infiltrating immune cells were recently demonstrated to be linked to the development of liver fibrosis.^14^ The macrophage compartment of the liver, traditionally called ‘Kupffer cells’, is constantly replenished to a significant extent by blood monocytes^15^ and is greatly augmented by an overwhelming number of infiltrating monocytes upon acute or chronic liver injury.^14^

In the present work, a marked increase in the surface expression of CD11a was noticed in both groups of patients with chronic hepatitis C liver disease compared to controls. In addition, a marked increase in the surface expression of CD11b and CD49d was also noticed in cirrhotic patients compared to those with CHC. These findings indicate that the progressive liver damage and the development of LC have a significant impact on integrins by intensifying of their expression on monocyte surfaces. The increase in surface expression provides the mechanism for endothelial transmigration required for hepatic infiltration by monocytes, as documented after inflammation and imply that a cell population of higher function may potentially appear.^16^,^17^ Those monocytes which respond to liver inflammatory environment with strongly increased adhesion properties would accumulate at the site of inflammation. Circulating monocytes are precursors of dendritic cells and macrophages of the liver.^18^ Activated by a liver inflammatory environment liver resident macrophages–Kupffer’s cells constitutively show high LFA-1, Mac-1, and VLA-4 expression that enable active participation in the intensification of inflammatory and immunological processes in the liver.^17^ Extensive mononuclear cell infiltration was found to be strongly correlated with liver damage in patients with chronic hepatitis B virus infection.^19^ Monocytes and activated macrophages contribute to the development and progression of fibrosis via expression of numerous cytokines, such as platelet-derived growth factor and transforming growth factor-β1, or generation of reactive oxygen intermediates.^20^ Participation of infiltrating monocytes in the development of liver damage and fibrosis in animal models is proved.^19^

Moreover, data revealed a marked increase in the circulating levels of soluble adhesive molecules sE-selectin, sICAM-1 and sVCAM-1 in both groups of patients with chronic hepatitis C liver disease compared to controls. Similar results are reported in patients with CLD,^21^ and CHC^10^,^22^ and the increased level was found to be correlated to the severity and progression of the disease. In addition, up-regulated ICAM-1 and VCAM-1 expression in the hepatic sinusoid cells, vascular endothelial cells, and hepatocytes were reported in chronic liver disease in several studies.^23^–^25^ Circulating adhesion molecules is the proteolytically released form of membrane-bound activated molecule.^26^ The activation of endothelial cells has a crucial role in modulation of leukocyte functions and mediating the transmigration of inflammatory cells, which induce inflammation, tissue damage and liver cirrhosis..^27^,^28^ These studies are in accordance with our findings which show that the increased concentrations of each of sICAM-1 and sVCAM-1 in patients with CHC and LC are strongly correlated with the increased surface integrins expression on monocyte in these patients. Endothelial cell incubation with antibodies against integrin receptors inhibits monocyte adhesion.^29^ The administration of anti-LFA-1 or anti-ICAM-1 antibodies diminishes significantly the stage of hepatocyte damage.^17^ Endothelial soluble adhesive molecules, induced by pro-inflammatory cytokines, are the markers of the functional condition of endothelial cells and probably reflect enodothelial activation.^30^,^31^

Our results also revealed marked elevation in circulating levels of ICAM-1 and VCAM-1 in cirrhotic patients compared to non-cirrhotic cases. Enhanced levels of these adhesion molecules may be the consequence of persistent activation of vascular endothelial cells which are able to produce connective tissue growth factor, a highly profibrogenic molecules involved in several fibrotic disorders, including those of the liver.^32^ Such elevation in circulating ICAM-1 and VCAM-1 levels may be also attributed to increased levels of TNF-α which was also reported in our study. Increased peripheral blood monocytes activity and elevated expression of TNF-α which was found to be correlated with liver disease activity (Child-Pugh stage B and C) were reported.^33^ TNF-α is a potent mediator of inflammation and sepsis^34^ and has a pleiotropic effect on a wide variety of cells including endothelial cells.^35^ Furthermore, data revealed the circulating levels of TNF-α in both groups of patients were strongly correlated with each of sICAM-1 and sVCAM-1 values in these patients. These findings indicate that TNF-α might directly induce the expression of ICAM-1 and VCAM-1 in vascular endothelial cells.^36^ Moreover, correlation was found between monocyte adhesion molecules CD11a, CD11b and CD49d and each of sICAM-1 and sVCAM-1 which suggest the useful use of these soluble markers as an indicator of enhanced monocyte recruitment to the liver.

Monocyte chemoattractant protein-1 is also implicated in the process of hepatic inflammation, recruiting monocytes and lymphocytes during liver injury. MCP-1 also activates directly hepatic stellate cells, which play a major role in hepatic fibrosis. The decrease in the circulating levels of MCP-1 in patients with CLD may be attributed to its increased utilization or its temporal expression and secretion in sites of inflammations which may suggest that MCP-1 is predominantly locally produced within the liver. In chronic hepatitis, MCP-1 expression was directly correlated with the degree of inflammatory infiltrate in the portal tract, activated stellate cells and monocyte/macrophages. In active cirrhosis, MCP-1 expression was present in the portal tract, epithelial cells of regenerating bile ducts, and the active septa surrounding regenerating nodules.^37^ There was a direct relationship between MCP-1 expression and monocyte infiltration after acute liver injury.^38^ MCP-1 level was found to be higher in hepatic veins than in peripheral blood and occurred in severe cases of liver diseases.^39^ However, our result revealed that the circulating levels of MCP-1 in controls and diseased groups were comparable.

## Conclusion

Monocyte/endothelial cell interactions and immune activation are features of chronic HCV infection and suggest their functional contribution to the perpetuation of intrahepatic inflammation and liver cirrhosis. Monocytes and activated macrophages contribute to the development and progression of fibrosis via expression of numerous cytokines. The modulation of monocyte-subset recruitment into the liver *via* adhesion molecules or chemokines/chemokine receptors and their subsequent differentiation may represent promising approaches for therapeutic interventions in human liver fibrosis. Moreover, the differential release of sE-selectin, sICAM-1 and sVCAM-1 in HCV-induced chronic liver disease suggest that adhesion molecules may influence the nature of the inflammatory cellular infiltrate and affect disease progression. Thus, measurement of serum soluble adhesion molecules may be useful for monitoring progression or regression of liver inflammation and fibrosis during chronic HCV infection especially since ALT values may only reflect hepatocellular necrosis and follow-up liver biopsies are contraindicated.

## Figures and Tables

**Table 1:** Clinicolaboratory data in patients with chronic hepatitis C liver disease.

Group (n=15)	Controls	CHC	LC
**Jaundice**	0 (0%)	0 (0%)	6 (20%)
**Palmer erythema**	0 (0%)	0 (0%)	15 (50%)
**Spider nevi**	0 (0%)	0 (0%)	14 (46.7%)
**Lower limbedema**	0 (0%)	0 (0%)	16 (53.3%)
**Hepatomegaly**	0 (0%)	14 (46.7%)	12 (40%)
**Ascites**	0 (0%)	3 (10%)	16 (53.3%)
**AST(IU/l)**	29.0±4.1	43.5±24.2	83.6±84.6
**ALT (IU/l)**	33.9±18.8	41.0±8.3	48.2±48.2
**Bilirubin (mg/dl)**	0.5±0.2	1.0±0.4	2.9±1.7
**Albumin (g/dl)**	4.2±0.3	3.9±0.5	2.1±0.3
**PT (sec)**	12.4±0.5	15.8±1.4	21.4±4.8

Data were expressed as means ± SD

**Table 2:** Laboratory findings in patients with chronic hepatitis C liver disease.

Group (n=15)	Controls	CHC	LC
**CD11a (%)**	78.69±25.53	88.40±9.96*	94.15±9.82**
**CD 11b (%)**	74.53±16.11	73.58±12,33	84.48±9.39*#
**CD49d (%)**	29.80±7.40	32.60±8.51	48.49±18.77**#
**sE-selectin (ng/ml)**	42.12±20.26	102.90±47.42**	109.49±50.36**
**sICAM-1 (ng/ml)**	201.53±83.96	697.57±429.80**	1027.97±398.18**#
**sVCAM-1 (ng/ml)**	344.61±29.36	1361.33±27.90**	1787.31±14.51**#
**TNF-α (pg/ml)**	11.73±4.02	36.47±17.81**	77.43±42.99**#
**IL-1β (pg/ml)**	1.36±0.43	1.90±0.99	1.67±0.76
**MCP-1 (pg/ml)**	50.78±31.12	58.34±36.45	66.43±47.03

Data expressed as means ± SE.

* ***p*** < 0.05,

** ***p*** < 0.01: Relative to Controls

# ***p*** < 0.01: Relative to CHC

**Table 3:** Correlations analyses in patients with chronic hepatitis C liver disease

Parameter	Correlation coefficient (*r*)	*p* value
**CD11a*vs.*sICAM-1**	0.317	>0.01
**CD11a*vs.*sVCAM-1**	0.366	> 0.01
**CD11b*vs.*sICAM-1**	0.251	> 0.05
**CD11b*vs*. sVCAM-1**	0.258	> 0.05
**CD49d*vs.*sICAM-1**	0.315	>0.01
**CD49d*vs.*sVCAM-1**	0.419	> 0.01
**sICAM-1*vs.*TNF-α**	0.355	>0.01
**sVCAM-1*vs.*TNF-α**	0.419	> 0.01
